# Relationships between area-level socioeconomic status and urbanization with active transportation, independent mobility, outdoor time, and physical activity among Canadian children

**DOI:** 10.1186/s12889-019-7420-y

**Published:** 2019-08-09

**Authors:** Christine Delisle Nyström, Joel D. Barnes, Sébastien Blanchette, Guy Faulkner, Geneviève Leduc, Negin A. Riazi, Mark S. Tremblay, François Trudeau, Richard Larouche

**Affiliations:** 1 Healthy Active Living and Obesity (HALO) Research Group, CHEO Research Institute, 401 Smyth Road, Ottawa, ON K1H 8L1 Canada; 2 Départment des sciences de l’activité physique, Université du Québec à Trois-Rivières, Quebéc, G9A 5H7 Canada; 3 School of Kinesiology, University of British Columbia, Vancouver, British Columbia V6T 1Z1 Canada; 4 Faculty of Health Sciences, University of Lethbridge, Lethbridge, Alberta T1K 3M4 Canada; 5 Department of Biosciences and Nutrition, Karolinska Institutet, NEO, 141 83 Huddinge, Sweden

**Keywords:** Active travel, Independent mobility, Moderate-to-vigorous physical activity, Steps, Social determinants of health, Time spent outdoors

## Abstract

**Background:**

Active transportation (AT), independent mobility (IM), and outdoor time are promising ways to increase children’s physical activity. However, in order to create interventions to increase those forms of physical activity, it is important to understand the relationships between area-level socioeconomic status (SES) and type of urbanization with AT, IM, outdoor time, and physical activity, and this was the aim of the study.

**Methods:**

One thousand six hundred ninety-nine children in grades 4 to 6 (mean age: 10.2 ± 1.0 years) from three Canadian regions participated. AT, IM, and outdoor time were assessed using questionnaires and physical activity was measured using the SC-StepRX pedometer. Area-level SES was assessed using the median household income of the census tract in which the school was located and type of urbanization was determined for each school using standardized procedures. Generalized linear and general linear mixed models were used to examine the relationships.

**Results:**

Area-level SES and the type of urbanization were generally not related to AT, IM, or physical activity for either gender. However, we observed that both boys and girls living in lower SES areas had decreased odds of spending > 2 h outdoors on weekend days compared to their peers from higher SES areas. Girls living in suburban or rural areas were more likely to spend > 2 h outdoors on weekdays compared to their urban counterparts.

**Conclusions:**

AT, IM, and physical activity are generally not associated with area-level SES or the type of urbanization in this sample of Canadian children. The finding regarding outdoor time showing that both boys and girls of lower SES areas had decreased odds of spending > 2 h outdoors on weekends compared to their peers from higher SES areas suggest that additional efforts should be implemented to offer outdoor play opportunities in lower SES areas.

**Electronic supplementary material:**

The online version of this article (10.1186/s12889-019-7420-y) contains supplementary material, which is available to authorized users.

## Background

Similar to many other developed countries, the majority of children/youth in Canada are not meeting the physical activity (PA) guidelines of 60 min of moderate-to-vigorous physical activity (MVPA) per day [1]. Low levels of PA and high levels of sedentary behaviour are well-established correlates in the development of childhood obesity [2]. Furthermore, there is evidence that high PA and low sedentary behaviour in children/youth are associated with better cardio-metabolic health [3] and mental health [4]. Thus, there is a need to find effective ways for children to increase their activity levels on a daily basis.

Active transportation (AT) is any self-propelled transport and is a very accessible and feasible way for children to increase their habitual PA. A systematic review found that children/youth who actively travel to school acquire more daily PA than inactive travellers [5]. Despite the health benefits associated with AT in children (e.g., reduced stress levels [6], lower incidence of metabolic syndrome [7]), AT has decreased considerably in many countries in recent decades [8, 9]. Independent mobility (IM), which refers to children’s ability to travel freely around their neighbourhood without adult supervision [10], may be one of the factors that is driving the decline in AT among children. Previous studies have found that IM is associated with AT [11], outdoor play [11, 12], and overall PA [13]. However, IM has declined markedly over the last few decades [10, 14, 15]. The amount of time spent outdoors has been found to be positively associated with habitual PA [16], MVPA, and step counts [17] in children. All these components are important as IM and AT may play critical roles in facilitating opportunities for engaging in outdoor time, and travelling to places like parks/playgrounds to play outdoors. However, in order to create interventions to increase PA, it is important to understand if or how social determinants such as area-level socioeconomic status (SES) and type of urbanization are related to AT, IM, outdoor time, and PA.

To date, few studies have investigated the relationship between area-level SES and AT, IM, outdoor time, and PA. Many studies have found that children from families owning fewer cars or with lower SES are more likely to use AT than children of more affluent families [18–20]. Veitch et al. [21] investigated the relationship between area-level SES and IM and found that children living in lower SES areas could visit more places without adult supervision in comparison to their peers from higher SES areas. With regard to PA, mixed results have been found with some studies finding significant differences between high and low SES and others not [22, 23]. Finally, for outdoor time one study found that children in lower SES households faced more restrictions regarding outdoor play than children from higher SES areas [23].

Additionally, a limited number of studies have examined the relationship between the type of urbanization and AT, IM, outdoor time, and PA. Studies conducted in Canada and the United States indicated a higher prevalence of AT or greater time spent engaging in AT among children living in urban areas [24, 25]. In contrast, Seliske et al. [26] reported that Canadian children/youth living in areas with greater urban sprawl were more likely to accumulate at least 30 min of daily AT. With regard to IM, some studies suggest that children living in rural areas have greater IM than their urban counterparts [27, 28], whereas others found no differences [29]. For PA, some studies have found that children from rural areas were more active than children from urban areas [30, 31], whereas another found the opposite [24]. To our knowledge, no study has investigated the relationship between outdoor time and the type of urbanization in children.

The aim of this study was to examine the relationships between area-level SES and the type of urbanization with measures of AT, IM, outdoor time, and PA in elementary school children living in urban, suburban, and rural areas within three different regions in Canada.

## Methods

### Study design and participants

The active transportation, independent mobility, and PA among school children (ATIM) study was a multi-site, cross-sectional study designed to assess AT, IM, and PA habits of Canadian children in grades 4–6. Data were collected between March 2016 and June 2017 in Vancouver, British Columbia; Ottawa, Ontario; and Trois-Rivières, Quebec. In each testing site a purposive sampling method was used to select schools located in census tracts with varying SES (as estimated by the median household income according to the 2006 Canadian census). A pool of interested schools was generated, and two lower SES and two higher SES schools were recruited in urban, suburban, and rural areas at each site. In Vancouver, an additional urban school was recruited, thus 37 schools participated. Overall, 1951 children agreed to take part in the study (54.2% response rate). Of these, 1699 children (87.1%) returned at least the child or parent questionnaire and were included in the analytical sample. The 1699 included children were more likely to be girls (x^2^ = 7.70; *p* = 0.006), reside in a suburban area (x^2^ = 11.10; *p* = 0.004) and reside in Trois-Rivières (x^2^ = 14.05; *p* = 0.001), and to have handed in their pedometer (x^2^ = 446.88; *p* < 0.001). There were no other differences observed between those included and excluded.

Parents/legal guardians provided written, informed consent and written assent was provided by the children. The ATIM project was approved by the Research Ethics Board at the Children’s Hospital of Eastern Ontario, University of British Columbia, and Université du Québec à Trois-Rivières as well as participating School Boards.

### ATIM protocol

Participating children and one of their parents/legal guardians were asked to complete separate questionnaires regarding their or their child’s travel behaviours and IM, respectively. The children also reported their age and gender, while the participating parent reported sociodemographic characteristics of the household. Starting the day after the distribution of the questionnaires, the child wore a pedometer for seven consecutive days to assess their PA. Both the child and parent questionnaires have been found to provide valid and reliable estimates of children’s AT and IM [32].

The children reported the number of active trips to and from various locations (e.g., school, friend’s home, parks/playgrounds) for each day of the week. The number of trips to and from all destinations were summed to come up with the participant’s total number of active trips over the seven-day measurement period.

Based on the six mobility licences proposed by Hillman [10] children were asked if they were allowed to do each of the following activities on their own: to cross main roads, travel to school, visit other places within walking distance, use public buses, cycle on main roads, and go out after dark. An IM index ranging from 0 to 6 was computed, with a score of 6 representing high IM [32].

Outdoor time was assessed in the parent questionnaire through two categorical questions: (i) ‘on a typical weekday, how much time does your child spend playing outdoors at the moment?’ and (ii) ‘on a typical weekend day, how much time does your child spend playing outdoors at the moment?’ For both questions the participating parent chose from the following five alternatives: ‘none’, ‘< 1 h’, ‘1–2 h’, ‘2–3 h’, and ‘> 3 h’. For all of the analyses the categories were condensed to ‘≤2 h’ or ‘> 2 h’. These questions have been found to provide reliable estimates regarding the amount of time children spend outside on weekdays and weekend days [33].

PA was objectively measured using sealed SC-StepRx pedometers (StepsCount, Deep River, ON, Canada). The participants wore the pedometers for seven consecutive days and were instructed to wear them at all times except when sleeping and during water-based activities. The SC-StepRX is a piezoelectric pedometer which provides valid measures of step counts and time spent in MVPA based on stepping frequency [34]. To be included in the analyses, participants had to have a minimum of three valid days (including one weekend day). These criteria were used because of the known variability between weekdays and weekend days in the literature [35]. Following Rowe et al.’s [36] rules, valid days were defined as those where between 1000 and 29,999 steps were recorded. Excluded days were replaced by the mean of valid weekdays or weekend days as appropriate. Daily step counts were then summed and divided by the number of valid days to provide the average number of steps per day. Finally, average MVPA per day was calculated by summing the number of minutes in MVPA per day, which was defined as > 110 steps/minute [34] and dividing it by the number of valid days. Whenever the step counts for a given day was deemed “invalid” as per Rowe et al.’s [36] rules, the MVPA data for that day was also considered invalid and the same approach was used to replace the MVPA data.

Area-level SES was classified as ‘low’ or ‘high’ based on the median household income of the 2006 census tract in which the school was located, relative to the average income for the region (i.e., census metropolitan area). Household-level SES was controlled for in all analyses and was assessed in the parent questionnaire by asking questions related to parental education (‘elementary school’, ‘secondary school’, ‘college’, ‘university’, or ‘graduate school’), home ownership (‘owned their home’ or ‘rented a home from Council or Housing Association’ or ‘privately rented’), and car ownership (‘no’, ‘yes, 1 car’ or ‘yes, 2 or more cars’). For the analyses, parental education, home ownership and car ownership were recoded to: ‘high school or less’, ‘college’, or ‘university’; ‘yes I own’ or ‘no I rent’; and ‘≤1 car’ or ‘≥2 cars’, respectively. Type of urbanization was classified as ‘urban’, ‘suburban’, or ‘rural’ according to the procedures described by Rainham et al. [24].

### Statistical analysis

Multiple imputation was done using the “mice” [37] package in R where 20 imputed datasets were generated with a maximum of 25 iterations per imputation. The predictive mean matching imputation method was used for continuous variables, the proportional odds model imputation method was used for ordered categorical variables, and the polytomous logistic regression method was used for unordered categorical variables. As recommended by Graham [38], the imputation model included all of the variables of interest in our analyses in addition to several auxiliary variables that may improve the prediction of missing values (Additional file 1).

Values are presented as means and standard deviations, and differences between girls and boys were determined using multivariate analysis of variance. The relationship between area-level SES and type of urbanization and the number of active trips per week were assessed using Poisson generalized linear mixed models and presented as an incidence rate ratio (IRR). An IRR represents the percent change in the dependent variable given a one-unit increase in the independent variable. The relationship between area-level SES and type of urbanization and IM, number of steps per day, and number of minutes of MVPA per day were assessed using general linear mixed models. Finally, the relationship between area-level SES and type of urbanization and the amount of time spent outside on weekdays and weekend days was assessed using logistic generalized linear mixed models. All continuous independent variables in all of the models were centered using the grand mean. School was used as the level two variable and the intercepts were allowed to vary. All analyses were conducted using estimates from the 20 imputed datasets and the supplementary material in Additional file 2 presents the results for the complete cases only. RStudio 1.1.442 (Boston: RStudio, Inc.) was used for all analyses.

## Results

The descriptive characteristics of the 1699 participants are shown in Table 1. Overall, the participants had a mean age of 10.2 ± 1.0 years and 55% were girls. In comparison to boys, girls had statistically significantly fewer IM licences, steps per day, and minutes of MVPA per day.

**Table 1:** Descriptive characteristics of the participants

	All (*n* = 1699)^a, b, c^		Girls (*n* = 935)		Boys (*n* = 764)			
	Mean ± SD	Range	Mean ± SD	Range	Mean ± SD	Range	*p*	Cohen’s *d*
Age (years)	10.2 ± 1.0	8–13	10.2 ± 1.0	8–13	10.2 ± 1.0	8–13	0.258	0.06
Number of active trips per week	11.4 ± 11.5	0–150	11.0 ± 11.1	0–150	11.8 ± 11.9	0–142	0.190	0.06
Independent mobility licences	2.2 ± 1.6	0–6	2.1 ± 1.6	0–6	2.3 ± 1.6	0–6	0.003	0.14
Steps per day	12,052 ± 3778	3370–24,886	11,387 ± 3452	3370–24,458	12,865 ± 3997	3449–24,886	< 0.001	0.40
MVPA (min/day)	65.2 ± 23.1	12–177	61.2 ± 20.4	12–151	70.0 ± 25.2	14–177	< 0.001	0.38
Outdoor time (weekdays)								
≤ 2 h per day	1373 (80.8%)	–	767 (82.0%)	–	606 (79.3%)	–	–	–
> 2 h per day	326 (19.2%)	–	168 (18.0%)	–	158 (20.7%)	–	–	–
Outdoor time (weekend days*)*								
≤ 2 h per day	832 (49.0%)	–	478 (51.1%)	–	354 (46.3%)	–	–	–
> 2 h per day	867 (51.0%)	–	457 (48.9%)	–	410 (53.7%)	–	–	–

*SD* Standard deviation, *MVPA*, *p P*-value, Moderate-to-vigorous physical activity

^a^The percentage of participating children from each site: Ottawa (Boys: 24.1%; Girls: 27.4%); Trois-Rivières (Boys: 31.4%; Girls: 28.7%); and Vancouver (Boys: 44.5%; Girls: 44.0%)

^b^The percentage of participating children from each type of urbanization: Urban: (Boys: 45.3%; Girls: 43.9%); Suburban (Boys: 31.9%; Girls: 30.9%); and Rural: (Boys: 22.8%; Girls: 25.2%)

^c^The percentage of participating children from high and low socioeconomic status: High (Boys: 56.3%; Girls: 59.5%) and Low: (Boys: 43.7%; Girls: 40.5%)

Table 2 shows the relationship between area-level SES and type of urbanization and the number of AT trips per week. For both boys and girls there were no statistically significant relationships between the number of active trips with area-level SES or type of urbanization. However, both boys and girls whose parents owned ≥2 cars reported fewer AT trips per week on average compared to their peers whose parents owned ≤1 car (IRR: 0.80; 95% confidence interval (CI): 0.77–0.84 for girls and 0.76–0.84 for boys).

**Table 2:** Relationship between area-level SES and type of urbanization with number of active trips per week

*Independent variables*	*Dependent variable: active trips per week*					
	Girls			Boys		
	*IRR*	*95% CI*	*p*	*IRR*	*95% CI*	*p*
Fixed Effects						
(Intercept)	12.28	9.13–16.53	**< 0.001**	9.25	6.86–12.46	**< 0.001**
School SES: low (ref: high)	1.09	0.86–1.38	0.473	0.94	0.75–1.18	0.614
Site: Vancouver (ref: Ottawa)	1.05	0.78–1.41	0.741	1.34	0.99–1.80	0.055
Site: Trois-Rivières	1.10	0.81–1.49	0.530	1.30	0.96–1.76	0.087
Type of urbanization: suburban (ref: urban)	0.89	0.67–1.19	0.447	1.16	0.87–1.55	0.303
Type of urbanization: rural	0.83	0.62–1.11	0.209	1.14	0.85–1.52	0.390
Parental education: high school or less (ref: University)	0.88	0.82–0.94	**< 0.001**	1.05	0.97–1.13	0.224
Parental education: college	0.98	0.93–1.03	0.448	1.11	1.06–1.18	**< 0.001**
Age in years (centered)	1.05	1.03–1.08	**< 0.001**	1.01	0.98–1.03	0.490
Car ownership: ≥ 2 cars (ref: ≤ 1 car)	0.80	0.77–0.84	**< 0.001**	0.80	0.76–0.84	**< 0.001**
Home ownership: yes (ref: no)	1.01	0.96–1.06	0.620	1.03	0.98–1.09	0.248
Random Effects						
τ_00, School_ID_	0.135			0.136		
N_School_ID_	37			37		
ICC_School_ID_	0.119			0.120		
Observations	935			764		
Tjur’s D	1.093			1.239		
AIC	7674.815			6483.770		
-2 Log-Likelihood	7650.815			6459.770		
Deviance	7485.331			6299.711		

*IRR* Incidence rate ratio, *CI* Confidence interval, *p P*-value, *SES* Socioeconomic status; τ_00_ variance within the dependent variable between schools, *N* Number of schools, *ICC* Intraclass correlation coefficient, *AIC* Akaike information criterion

No statistically significant relationship was observed between area-level SES and type of urbanization and the number of IM licences (Table 3). For girls, participants from Trois-Rivières had greater IM than their counterparts from Ottawa (β = 0.67; 95% CI: 0.28–1.07), whereas no difference in IM was observed between girls from Ottawa and Vancouver (β = − 0.22; 95% CI: − 0.59 - 0.15). Furthermore, for every 1 year increase in age, the number of IM licences increased by 0.55 (95% CI: 0.45–0.65) in girls and 0.63 (95% CI: 0.52–0.75) in boys on average.

**Table 3:** Relationship between area-level SES and type of urbanization with the number of independent mobility licences

*Independent variables*	*Dependent variable: independent mobility licences*					
	Girls			Boys		
	*B*	*95% CI*	*p*	*B*	*95% CI*	*p*
Fixed Effects						
(Intercept)	1.85	1.45–2.25	**< 0.001**	2.23	1.82–2.63	**< 0.001**
School SES: low (ref: high)	−0.19	−0.50 - 0.12	0.239	0.05	− 0.25 - 0.35	0.725
Site: Vancouver (ref: Ottawa)	−0.22	− 0.59 - 0.15	0.248	− 0.18	− 0.55 - 0.18	0.330
Site: Trois-Rivières	0.67	0.28–1.07	**0.002**	0.36	−0.03 - 0.76	0.076
Type of urbanization: suburban (ref: urban)	0.01	−0.36 - 0.37	0.976	−0.20	−0.56 - 0.15	0.261
Type of urbanization: rural	0.14	−0.24 - 0.52	0.466	0.05	−0.32 - 0.43	0.778
Parental education: high school or less (ref: University)	0.07	−0.23 - 0.37	0.635	−0.24	−0.60 - 0.12	0.197
Parental education: college	0.18	−0.05 - 0.41	0.131	0.00	−0.26 - 0.26	0.992
Age in years (centered)	0.55	0.45–0.65	**< 0.001**	0.63	0.52–0.75	**< 0.001**
Car ownership: ≥ 2 cars (ref: ≤ 1 car)	−0.00	−0.20 - 0.19	0.961	−0.16	− 0.40 - 0.07	0.186
Home ownership: yes (ref: no)	0.07	−0.16 - 0.29	0.567	0.28	0.02–0.55	**0.041**
Random Effects						
σ^2^	1.834			2.109		
τ_00, School_ID_	0.138			0.097		
N_School_ID_	37			37		
ICC_School_ID_	0.070			0.044		
Observations	935			764		
R^2^ / Ω_0_^2^	0.294 / 0.293			0.243 / 0.241		
AIC	3283.974			2787.764		
Deviance	3257.974			2761.764		

*B* Unstandardized beta coefficient, *CI* confidence interval, *p p*-value, *SES* Socioeconomic status, σ^2^ variance within cluster groups (schools), τ_00_ variance within the dependent variable between schools, *N* Number of schools, *ICC* Intraclass correlation coefficient, R^2^ / Ω_0_^2^ pseudo R^2^ estimates, *AIC* Akaike information criterion

The relationships between area-level SES and type of urbanization with outdoor time are presented for weekdays in Table 4 and weekend days in Table 5. For girls, those who lived in a suburban or rural area compared to an urban area had 2.10 and 2.04 greater odds of spending > 2 h outside on weekdays (95% CI: 1.24–3.56 and 1.17–3.58, respectively). For boys, on weekdays, there were no statistically significant relationships between area-level SES and type of urbanization with the amount of outdoor time. For weekend day outdoor time, girls and boys who lived in lower SES areas compared to higher SES areas were at decreased odds of spending > 2 h outside (girls odds ratio 0.48; 95% CI: 0.32–0.71; boys odds ratio: 0.63; 95% CI: 0.41–0.97). For girls, those who lived in a rural area compared to an urban area had 2.34 greater odds of spending > 2 h outside on weekend days (95% CI: 1.44–3.79). No statistically significant relationships were found between type of urbanization and amount of time spent outside on weekend days among boys.

**Table 4:** Relationship between area-level SES and type of urbanization with weekday outdoor time

*Independent variables*	*Dependent variable: weekday outdoor time (≤2 h (ref)* vs. *> 2 h)*					
	Girls			Boys		
	*Odds Ratio*	*95% CI*	*p*	*Odds Ratio*	*95% CI*	*p*
Fixed Effects						
(Intercept)	0.11	0.06–0.21	**< 0.001**	0.18	0.11–0.31	**< 0.001**
School SES: low (ref: high)	0.92	0.59–1.44	0.717	1.11	0.78–1.60	0.555
Site: Vancouver (ref: Ottawa)	1.43	0.83–2.43	0.194	1.38	0.87–2.18	0.175
Site: Trois-Rivières	1.26	0.69–2.30	0.460	0.94	0.56–1.60	0.821
Type of urbanization: suburban (ref: urban)	2.10	1.24–3.56	**0.006**	1.01	0.67–1.54	0.957
Type of urbanization: rural	2.04	1.17–3.58	**0.013**	1.07	0.66–1.74	0.775
Parental education: high school or less (ref: University)	1.11	0.62–1.99	0.728	1.51	0.85–2.69	0.164
Parental education: college	1.74	1.13–2.67	**0.011**	1.19	0.77–1.84	0.423
Age in years (centered)	0.86	0.71–1.04	0.127	0.91	0.76–1.10	0.330
Car ownership: ≥ 2 cars (ref: ≤ 1 car)	1.02	0.70–1.50	0.917	0.90	0.61–1.32	0.582
Home ownership: yes (ref: no)	0.79	0.52–1.21	0.287	1.17	0.75–1.82	0.488
Random Effects						
τ_00, School_ID_	0.140			0.000		
N_School_ID_	37			37		
ICC_School_ID_	0.041			0.000		
Observations	935			764		
Tjur’s D	0.050			0.010		
AIC	872.980			794.820		
-2 Log-Likelihood	848.980			770.820		
Deviance	822.965			770.820		

*CI*, Confidence interval, *p p*-value, *SES* Socioeconomic status, *N* Number of schools, τ_00_ variance within the dependent variable between schools, *ICC* Intraclass correlation coefficient, *AIC* Akaike information criterion

**Table 5:** Relationship between area-level SES and type of urbanization in weekend day outdoor time

*Independent variables*	*Dependent variable: weekend day outdoor time (≤2 h (ref)* vs. *> 2 h)*					
	Girls			Boys		
	*Odds Ratio*	*95% CI*	*p*	*Odds Ratio*	*95% CI*	*p*
Fixed Parts						
(Intercept)	1.05	0.63–1.73	0.863	0.78	0.44–1.40	0.411
School SES: low (ref: high)	0.48	0.32–0.71	**< 0.001**	0.63	0.41–0.97	**0.034**
Site: Vancouver (ref: Ottawa)	0.73	0.47–1.15	0.174	0.83	0.49–1.40	0.491
Site: Trois-Rivières	1.63	0.97–2.74	0.065	2.08	1.16–3.72	**0.014**
Type of urbanization: suburban (ref: urban)	1.36	0.87–2.13	0.177	0.76	0.46–1.26	0.291
Type of urbanization: rural	2.34	1.44–3.79	**< 0.001**	1.45	0.84–2.51	0.187
Parental education: high school or less (ref: University)	1.22	0.77–1.95	0.398	1.68	0.99–2.87	0.057
Parental education: college	1.53	1.08–2.19	**0.018**	1.52	1.04–2.23	**0.032**
Age in years (centered)	0.76	0.65–0.88	**< 0.001**	0.93	0.79–1.10	0.421
Car ownership: ≥ 2 cars (ref: ≤ 1 car)	0.86	0.63–1.16	0.321	1.23	0.87–1.74	0.231
Home ownership: yes (ref: no)	0.83	0.59–1.17	0.285	1.32	0.90–1.95	0.156
Random Parts						
τ_00, School_ID_	0.137			0.183		
N_School_ID_	37			37		
ICC_School_ID_	0.040			0.053		
Observations	935			764		
Tjur’s D	0.102			0.108		
AIC	1242.057			1014.855		
-2 Log-Likelihood	1218.057			990.855		
Deviance	1182.387			953.839		

*CI* confidence interval, *p p*-value, *SES* Socioeconomic status, τ_00_ variance within the dependent variable between schools, *N* Number of schools, *ICC* Intraclass correlation coefficient, *AIC* Akaike information criterion

No statistically significant relationship was found between area-level SES and type of urbanization with the average number of steps per day (Table 6) and average minutes of MVPA per day (Table 7) in either girls or boys. In boys only, with every 1 year increase in age, average steps and minutes of MVPA decreased (β = − 478.02; 95% CI: − 778.04 - -178.00 and β = − 3.67; 95% CI: − 5.55 - -1.79, respectively).

**Table 6:** Relationship between area-level SES and type of urbanization with average number of steps per day

*Independent variables*	*Dependent variable: steps per day*					
	Girls			Boys		
	*B*	*95% CI*	*p*	*B*	*95% CI*	*p*
Fixed Effects						
(Intercept)	11,238.91	10,027.93–12,449.88	**< 0.001**	12,811.19	11,529.94–14,092.44	**< 0.001**
School SES: low (ref: high)	− 331.50	− 1291.55 - 628.56	0.502	− 380.06	− 1357.45 - 597.32	0.449
Site: Vancouver (ref: Ottawa)	518.26	− 630.67 - 1667.18	0.381	− 144.87	− 1339.18 - 1049.45	0.813
Site: Trois-Rivières	− 165.61	− 1384.79 - 1053.57	0.791	301.87	− 955.33 - 1559.08	0.640
Type of urbanization: suburban (ref: urban)	266.78	− 876.33 - 1409.88	0.649	89.97	− 1072.09 - 1252.04	0.880
Type of urbanization: rural	−78.52	− 1250.97 - 1093.93	0.896	154.67	− 1060.84 - 1370.17	0.804
Parental education: high school or less (ref: University)	217.16	− 501.66 - 935.98	0.556	−459.46	− 1406.80 - 487.87	0.346
Parental education: college	93.23	−452.51 - 638.96	0.739	− 397.81	− 1081.85 - 286.23	0.259
Age in years (centered)	−56.42	− 288.97 - 176.12	0.636	−478.02	−778.04 - -178.00	**0.003**
Car ownership: ≥ 2 cars (ref: ≤ 1 car)	136.17	− 328.47 - 600.81	0.568	185.22	− 432.94 - 803.38	0.559
Home ownership: yes (ref: no)	−65.26	−597.69 - 467.16	0.811	183.09	− 523.90 - 890.08	0.614
Random Effects						
σ^2^	10,316,244.723			14,336,948.928		
τ_00, School_ID_	1,673,547.941			1,455,090.348		
N_School_ID_	37			37		
ICC_School_ID_	0.140			0.092		
Observations	935			764		
R^2^ / Ω_0_^2^	0.167 / 0.160			0.140 / 0.129		
AIC	17,836.433			14,823.427		
Deviance	17,810.433			14,797.427		

*B* Unstandardized beta coefficient, *CI* Confidence interval, *p p*-value, *SES* Socioeconomic status, *σ*^*2*^ variance within cluster groups (schools), τ_00_ variance within the dependent variable between schools, *N* Number of schools, *ICC* Intraclass correlation coefficient, R^2^ / Ω_0_^2^ pseudo R^2^ estimates, *AIC* Akaike information criterion

**Table 7:** Relationship between area-level SES and type of urbanization with average minutes of MVPA per day

*Independent variables*	*Dependent variable: MVPA per day*					
	Girls			Boys		
	*B*	*95% CI*	*p*	*B*	*95% CI*	*p*
Fixed Effects						
(Intercept)	58.45	51.51–65.38	**< 0.001**	67.52	59.31–75.74	**< 0.001**
School SES: low (ref: high)	−1.13	−6.63 - 4.36	0.687	−1.54	−7.83 - 4.74	0.632
Site: Vancouver (ref: Ottawa)	4.73	− 1.83 - 11.28	0.163	0.96	−6.72 - 8.64	0.806
Site: Trois-Rivières	0.57	−6.40 - 7.55	0.873	4.54	−3.53 - 12.61	0.275
Type of urbanization: suburban (ref: urban)	1.05	−5.48 - 7.57	0.754	1.07	−6.41 - 8.56	0.779
Type of urbanization: rural	−0.07	−6.78 - 6.63	0.983	2.50	−5.31 - 10.30	0.533
Parental education: high school or less (ref: University)	2.05	−2.22 - 6.32	0.352	−2.85	−8.77 - 3.08	0.351
Parental education: college	0.68	−2.56 - 3.92	0.683	−3.66	−7.94 - 0.62	0.099
Age in years (centered)	−1.26	−2.64 - 0.12	0.080	−3.67	−5.55 - -1.79	**< 0.001**
Car ownership: ≥ 2 cars (ref: ≤ 1 car)	0.98	−1.78 - 3.74	0.490	1.45	−2.42 - 5.31	0.467
Home ownership: yes (ref: no)	−0.48	−3.64 - 2.68	0.769	0.66	−3.77 - 5.09	0.771
Random Effects						
σ^2^	364.542			560.441		
τ_00, School_ID_	53.241			62.024		
N_School_ID_	37			37		
ICC_School_ID_	0.127			0.100		
Observations	935			764		
R^2^ / Ω_0_^2^	0.160 / 0.153			0.153 / 0.144		
AIC	8249.166			7071.202		
Deviance	8223.166			7045.202		

*MVPA* Moderate-to-vigorous physical activity, *B* Unstandardized beta coefficient, *CI* Confidence interval, *p P*-value, *SES* Socioeconomic status, *σ*^*2*^ variance within cluster groups (schools), *τ*_*00*_ variance within the dependent variable between schools, *ICC* Intraclass correlation coefficient, *R*^*2*^ */ Ω*_*0*_^*2*^ pseudo R^2^ estimates, *AIC* Akaike information criterion

## Discussion

The aim of this study was to examine the relationships between area-level SES and type of urbanization with measures of AT, IM, outdoor time, and PA while controlling for household level SES. Overall, area-level SES and the type of urbanization were not associated with AT, IM, or PA for girls or boys. However, we observed that both boys and girls of lower SES areas had decreased odds of spending > 2 h outdoors on weekend days compared to their peers from higher SES areas. Girls living in suburban or rural areas were also more likely to spend > 2 h outdoors on weekdays compared to their urban counterparts.

### Active transportation, socioeconomic status, and type of urbanization

A few studies have investigated the relationship between AT and SES in children. Two studies found that children from less affluent households were more likely to use AT than their more affluent peers [18, 39]. Another study found that children who live in low SES neighborhoods reported more AT to/from school than children from high SES areas [19]. In the present study we found no evidence that area-level SES was related to the number of active trips the children took. A couple of the aforementioned studies [18, 19] hypothesized that one of the reasons why children from lower SES areas have greater AT is that cars are less accessible, which is supported by our results. We found that girls and boys whose parents owned ≥2 cars reported fewer AT trips per week on average than their peers whose parents owned ≤1 car. Interestingly, two European studies have found that children who reside in more deprived areas were less likely to walk or cycle to school in comparison to their peers who live in higher SES areas [40, 41]. Aarts et al. [40] attributed the lower use of AT in low SES areas to neighbourhood characteristics such as perceived social cohesion and social safety. It could be hypothesized that the null findings in the present study regarding area-level SES could be due to the fact that regardless of SES level, parents overall perceive more danger in their neighbourhood now than 10 to 15 years ago (when the majority of other studies collected data), thus limiting their child’s AT. Another possibility could be that car ownership has increased in low income areas in the past few decades. It is also important to note that the previous studies usually focused on the trip to/from school, whereas the present study included a range of destinations.

We found no difference in the number of active trips between children living in urban, suburban, and rural areas. These findings are opposite to those of previous studies that examined AT beyond the trip to/from school among American children/adolescents [25] and adolescents in Nova Scotia, Canada [24]. The difference found between the two studies could be due to when the data were collected, 2017 in the present study and in 2001 in the study by Yang et al. [25] as it has been found that several countries are observing a consistent decline in AT [8]. Furthermore, the previous studies used different methodologies: Yang et al. [25] used telephone interviews and Rainham et al. [24] used global positioning systems to determine the amount of time spent engaging in AT.

### Independent mobility, socioeconomic status, and type of urbanization

In this study we found that IM was not influenced by area-level SES, which is consistent with a study by Schoeppe et al. [42]. The only significant difference observed for IM was between the sites. We found that girls from Trois-Rivières had 0.67 (*p* = 0.002) more IM licences than their peers from Ottawa. Ottawa is a larger city (1.38 million) than Trois-Rivières (159000) [43] and Canadians living in smaller cities tend to be less concerned about their personal safety from crime in comparison to residents of larger cities [44]. Parental perception of neighborhood street and sidewalk safety [45] and ‘stranger danger’ [46] have been found to be associated with IM. Therefore, one could speculate that parents in larger cities could perceive greater danger than those in smaller cities, and they may impose more restrictions on their children’s IM.

The absence of difference in IM between the type of urbanization concurs with findings from another study [29]. However, two other studies suggest that children living in rural areas have greater IM than their urban peers [27, 28]. The differences between studies could be due to other factors such as: where the study took place (e.g., Canada and Australia vs. Finland and Belarus), the degree of urban density in the cities investigated, the layout of the city, or changes over time, as the other studies were done 10–15 years earlier.

### Outdoor time, socioeconomic status, and type of urbanization

To the best of our knowledge, this is the first study investigating the relationship between outdoor time with area-level SES and the type of urbanization. We found that children living in lower SES areas had decreased odds of spending > 2 h outside on weekends compared to their peers from higher SES areas. A study by Dumoid et al. [47] found that children living in higher SES households had greater access to outdoor play equipment than children living in lower SES households. From a different perspective, Tandon et al. [23] reported that children in lower SES households faced more restrictions regarding outdoor play than children from higher SES households. Therefore, our results could reflect greater access to outdoor play equipment and/or more freedom to play outside among children in higher SES areas.

We observed that girls living in rural areas had statistically significantly greater odds of spending > 2 h outside on weekdays and weekend days than their peers living in urban areas. A similar, but non-significant trend was observed in boys. A study by Matz et al. [48] found that rural Canadian children spent approximately 0.7 more hours outside than their urban counterparts, which they hypothesized was due to greater outdoor play and chores. These results could also be due to the fact that there is more room to play in rural areas and that these areas are perceived as safer by parents.

### Physical activity, socioeconomic status, and type of urbanization

To date mixed results have been observed regarding area-level SES and PA. In the present study we found that area-level SES was not related to step counts or the amount of time spent in MVPA for girls or boys. This is consistent with another study who found that the amount of time spent in MVPA was not statistically different for children of low, middle, or high SES households [23]. However, a study by McCormack et al. [22] found that children in higher SES areas had greater odds of meeting the daily step count recommendations. With regard to type of urbanization, we found no differences for average steps or MVPA per day. This is inconsistent with the literature where studies have found that rural children have greater PA scores [31] and are more likely to be active [30] than children residing in urban areas. However, Rainham et al. [24] reported that youth living in rural areas in Nova Scotia were less active than those living in urban and suburban areas. Therefore, due to the inconsistencies in the literature, future studies investigating the relationship between area-level SES, the type of urbanization, and PA in children are warranted.

### Strengths, limitations, and implications

This study was strengthened by its large sample size as well as the use of purposive sampling in order to include children from high and low SES areas as well as from urban, suburban, and rural areas, in three different regions of Canada. Additional strengths include the use of validated questionnaires to assess AT and IM [32] as well as the use of validated pedometers [34]. The inclusion of a range of destinations in the assessment of AT was an additional strength, as the majority of studies to date have just investigated AT in relation to trips to/from school. This study was limited by its cross-sectional design, which limits the ability to make causal inferences as well as its low response rate (54.2%). Additionally, the large amount of missing data was also a limitation that must be acknowledged. However, it is important to highlight that similar results were found when using multiple imputation and complete cases (Additional file 2). Another limitation was that AT, IM, and outdoor time were assessed by self- or proxy-reports and as such, these measures may be vulnerable to social desirability and recall bias. Finally, as recruitment only occurred in three regions across Canada, the results may not be generalizable to all Canadian children.

It is important to acknowledge that the effect of area-level SES and type of urbanization may differ between countries. For example, developing countries are undergoing a rapid PA transition characterized by a shift from a lifestyle associated with high energy expenditure to a modern and more sedentary Western lifestyle [49]. In general, this shift is first observed in rapidly growing urban areas and then in suburban and rural areas. The transition is typically associated with a decline in AT and PA [49–51]. This underscores the need for collecting local data to inform the development of context-specific PA interventions.

This paper has public health implications as it is well established that physical inactivity is associated with a higher risk of obesity, cardiovascular risk factors, and poor mental health [2–4]. Furthermore, previous research also indicates that children living in lower SES areas are at greater risk for obesity [52]. To counteract this risk, practitioners and policy-makers should ensure that PA opportunities are available for children living in low SES areas. Our observation that children attending schools in low SES areas were less likely to spend at least 2 h per day outside suggests that there may be a need for interventions in this area. For instance, schools could establish joint use agreements [53] with community partners so that facilities and equipment are available for children to play outside. Such interventions could also benefit children living in urban areas who spend less time outside.

## Conclusions

Overall, area-level SES and the type of urbanization were not associated with AT, IM, or PA for girls or boys. Due to the fact that the literature is conflicting, future studies should use a common protocol in order for researchers to determine whether the inconsistencies are related to measurement issues or other factors. We observed that both girls and boys of lower SES areas had decreased odds of spending > 2 h outdoors on weekends than their peers from higher SES areas. This finding suggests that additional efforts should be implemented to offer outdoor play opportunities in lower SES areas and in urban areas.

## Additional files

Additional file 1: List of all of the variables included in the imputation model. (DOCX 22 kb)

Additional file 2: Results from the complete case analyses. (DOCX 52 kb)

## Data Availability

The datasets supporting the conclusions of this article are available upon request to Dr. Richard Larouche (richard.larouche@uleth.ca).

## Abbreviations

AT: Active transportation
ATIM: Active transportation, independent mobility, and physical activity among school children study
IM: Independent mobility
IRR: Incidence rate ratio
MVPA: Moderate-to-vigorous physical activity
PA: Physical activity
SES: Socioeconomic status
