# Supplementary material for: Relationships between area-level socioeconomic status and urbanization with active transportation, independent mobility, outdoor time, and physical activity among Canadian children
Source: BMC Public Health. 2019 Aug 9;19:1082. doi: 10.1186/s12889-019-7420-y (PMC6688238; doi:10.1186/s12889-019-7420-y)
Supplement: Supplementary file 1 — List of all of the variables included in the imputation model. (DOCX 22 kb) [file 12889_2019_7420_MOESM1_ESM.docx]

**Multiple imputation model**

| **Variable** | **Role in model** | **Constraints** |
| --- | --- | --- |
| School_ID | Predictor | N/A |
| Site_ID | Predictor | N/A |
| Urban_Type (type of urbanization) | Predictor | N/A |
| SchoolSES | Predictor | N/A |
| CQ_gender | Predictor/imputed | N/A |
| CQ_age | Predictor/imputed | No decimals |
| CQ_grade | Imputed only (to avoid collinearity with age) | N/A |
| CQ9_ToSchoolLong (school travel time; categorical variable) | Predictor/imputed | N/A |
| CQ11_LikeTravel (preferred travel mode) | Predictor/imputed | N/A |
| CQ15_Bike (child has a bicycle) | Predictor/imputed | N/A |
| CQ19a_Safe (child feels safe in their neighbourhood) | Predictor/imputed | N/A |
| CQ20_Far_Own (home range alone) | Predictor/imputed | N/A |
| CQ20_Far_Friends (home range with friends) | Imputed only to avoid collinearity | N/A |
| PQ2_Days_Collected (number of days child is picked up by an adult at school) | Predictor/imputed | Integer between 0 and 7 |
| PQ23_Phone (child has a mobile phone) | Predictor/imputed | N/A |
| PQ25_WorriedTraffic (parent worried about traffic) | Predictor/imputed | N/A |
| PQ26_Far_Own (home range alone) | Imputed only to avoid collinearity | N/A |
| PQ27_Far_Friends (home range with friends) | Imputed only to avoid collinearity | N/A |
| PQ28_WeekOut (time spent outdoors on weekdays) | Predictor/imputed | N/A |
| PQ29_WeekendOut (time spent outdoors on weekend days) | Predictor/imputed | N/A |
| PQ30_Ill (child has illness, disability, infirmity) | Predictor/imputed | N/A |
| PQ31_ParentTravel (parent’s primary school travel mode) | Predictor/imputed | N/A |
| PQ32_ParentOwn (age at which parent was allowed to get around on their own | Predictor/imputed | Integer between 3 and 20 |
| PQ34_ParentWorkWalk (parent walks to work) | Imputed only to avoid collinearity | N/A |
| PQ34_ParentWorkBike (parent bikes to work) | Imputed only to avoid collinearity | N/A |
| PQ34_ParentWorkPub (parent uses public transit to get to work) | Imputed only to avoid collinearity | N/A |
| PQ34_ParentWorkCar (parent drives to work) | Predictor/imputed | N/A |
| PQ35_AdultLookout (most adults in the neighbourhood look out for other children) | Predictor/imputed | N/A |
| PQ35_Afraid (some people in the area make you afraid to let your child play outside) | Predictor/imputed | N/A |
| PQ36_Cars (car ownership) | Predictor/imputed | N/A |
| PQ42_AgeYou (age of parent; categorical variable) | Predictor/imputed | N/A |
| PQ43_GenderYou (gender of parent) | Predictor/imputed | N/A |
| PQ44_WorkYou (work of the parent) | Predictor/imputed | N/A |
| PQ46_Language (whether family speaks a language other than French or English at home) | Predictor/imputed | N/A |
| Steps_day (average steps per day) | Predictor/imputed | Integer between 1000 and 29 999 (following Rowe’s rules [36]) |
| MVPA_day (average minutes spent in MVPA) | Imputed only to avoid collinearity | ≥ 1 |
| CQ_ActiveT (number of trips to/from all destinations, child report) | Imputed only – this variable includes a lot of zeros and is highly skewed to the right | Integer between 0 and 175 |
| HighEdu (highest level of education in household, 3-level) | Predictor/imputed | N/A |
| CQ_IM_Index (independent mobility index) | Predictor/imputed | Integer between 0 and 6 |
| PQ_IM_Index (independent mobility index) | Imputed only to avoid collinearity | Integer between 0 and 6 |
| OwnHome (whether parents own their home) | Predictor/imputed | N/A |
| Distance AM (in meters) | Predictor/imputed | ≥ 1 |
| Distance PM (in meters) | Imputed only to avoid collinearity | ≥ 1 |
| Walkability400 (walkability index within 400m buffer) | Predictor/imputed | N/A |
| Walkability1600 (walkability index within 1600m buffer) | Imputed only to avoid collinearity | N/A |
| AST_volume (volume of AT to/from school, child report – in km/week) | Imputed only – this variable includes a lot of zeros and is highly skewed to the right | 0 ≤ AST volume ≤ 100 |
| PQ_all_trips (number of trips to/from all destinations, parent report) | Imputed only to avoid collinearity | Integer between 0 and 175 |
| MVPA_weekdays (average minutes in MVPA on weekdays) | Imputed only to avoid collinearity | ≥ 1 |
| MVPA_weekends (average minutes of MVPA on weekend days) | Imputed only to avoid collinearity | ≥ 1 |
| Steps_weekdays (average steps on weekdays) | Imputed only to avoid collinearity | Integer between 1000 and 29 999 (following Rowe’s rules [36]) |
| Steps_weekends (average steps on weekend days) | Imputed only to avoid collinearity | Integer between 1000 and 29 999 (following Rowe’s rules [36]) |
| PQ_AST_volume | Imputed only to avoid collinearity | 0 ≤ AST volume ≤ 100 |
| CQ_AST_trips (categorical variable for the number of AT trips to/from school) | Predictor/imputed | N/A |
| PQ_AST_trips (categorical variable for the number of AT trips to/from school) | Imputed only to avoid collinearity | N/A |

Note: variables that start with CQ are from the child questionnaire and those that start with PQ are from the parent questionnaire.

NA, Not applicable; SES, Socioeconomic status; MVPA, Moderate-to-vigorous physical activity; IM, Independent mobility; AST, Active school transport; AT, Active transport
