# Supplementary material for: Relationships between area-level socioeconomic status and urbanization with active transportation, independent mobility, outdoor time, and physical activity among Canadian children
Source: BMC Public Health. 2019 Aug 9;19:1082. doi: 10.1186/s12889-019-7420-y (PMC6688238; doi:10.1186/s12889-019-7420-y)
Supplement: Supplementary file 2 — Results from the complete case analyses. (DOCX 52 kb) [file 12889_2019_7420_MOESM2_ESM.docx]

**Table S1.** Relationship between area-level SES and type of urbanization with number of active trips per week.

| *Independent variables* | *Dependent variable: active trips per week* | | | | | | | |
| --- | --- | --- | --- | --- | --- | --- | --- | --- |
|  |  | Girls | | |  | Boys | | |
|  |  | *IRR* | *95% CI* | *p* |  | *IRR* | *95% CI* | *p* |
| **Fixed Effects** | | | | | | | | |
| (Intercept) |  | 12.02 | 9.03 - 15.99 | **<0.001** |  | 9.74 | 7.06 - 13.45 | **<0.001** |
| School SES: low (ref: high) |  | 1.09 | 0.87 - 1.37 | 0.437 |  | 0.86 | 0.67 - 1.10 | 0.229 |
| Site: Vancouver (ref: Ottawa) |  | 1.09 | 0.82 - 1.44 | 0.551 |  | 1.39 | 1.00 - 1.91 | **0.047** |
| Site: Trois-Rivières |  | 1.13 | 0.84 - 1.51 | 0.413 |  | 1.35 | 0.97 - 1.88 | 0.072 |
| Type of urbanization: suburban (ref: urban) |  | 0.94 | 0.71 - 1.24 | 0.639 |  | 1.26 | 0.92 - 1.72 | 0.147 |
| Type of urbanization: rural |  | 0.92 | 0.69 - 1.21 | 0.537 |  | 1.16 | 0.85 - 1.59 | 0.339 |
| Parental education: high school or less (ref: University) |  | 0.93 | 0.86 - 1.01 | 0.088 |  | 0.99 | 0.91 - 1.09 | 0.914 |
| Parental education: college |  | 0.93 | 0.88 - 0.99 | **0.023** |  | 1.12 | 1.06 - 1.19 | **<0.001** |
| Age in years (centered) |  | 1.06 | 1.04 - 1.09 | **<0.001** |  | 0.99 | 0.97 - 1.02 | 0.495 |
| Car ownership: ≥ 2 cars (ref: ≤ 1 car) |  | 0.79 | 0.76 - 0.83 | **<0.001** |  | 0.84 | 0.79 - 0.88 | **<0.001** |
| Home ownership: yes (ref: no) |  | 1.01 | 0.95 - 1.07 | 0.811 |  | 0.93 | 0.88 - 0.99 | **0.032** |
| **Random Effects** | | | | | | | | |
| τ_00, School_ID_ |  | 0.123 | | |  | 0.159 | | |
| N_School_ID_ |  | 37 | | |  | 37 | | |
| ICC_School_ID_ |  | 0.110 | | |  | 0.137 | | |
| Observations |  | 743 | | |  | 603 | | |
| Tjur's D |  | 0.567 | | |  | 0.645 | | |
| AIC |  | 6039.005 | | |  | 5140.724 | | |
| -2 Log-Likelihood |  | 6015.005 | | |  | 5116.724 | | |
| Deviance |  | 5860.337 | | |  | 4960.418 | | |

IRR, Incidence rate ratio; CI, Confidence interval; p, P-value; SES, Socioeconomic status; τ_00_, variance within the dependent variable between schools; N, Number of schools; ICC, Intraclass correlation coefficient- AIC; Akaike information criterion

**Table S2.** Relationship between area-level SES and type of urbanization with the number of independent mobility licences.

| *Independent variables* | *Dependent variable: independent mobility licences* | | | | | | | |
| --- | --- | --- | --- | --- | --- | --- | --- | --- |
|  |  | Girls | | |  | Boys | | |
|  |  | *B* | *95% CI* | *p* |  | *B* | *95% CI* | *p* |
| **Fixed Effects** | | | | | | | | |
| (Intercept) |  | 1.89 | 1.47 - 2.31 | **<0.001** |  | 2.29 | 1.85 - 2.72 | **<0.001** |
| School SES: low (ref: high) |  | -0.25 | -0.57 - 0.07 | 0.132 |  | 0.05 | -0.28 - 0.37 | 0.776 |
| Site: Vancouver (ref: Ottawa) |  | -0.23 | -0.61 - 0.15 | 0.239 |  | -0.27 | -0.67 - 0.12 | 0.182 |
| Site: Trois- Rivières |  | 0.74 | 0.33 - 1.15 | **<0.001** |  | 0.37 | -0.06 - 0.80 | 0.094 |
| Type of urbanization: suburban (ref: urban) |  | 0.09 | -0.29 - 0.47 | 0.645 |  | -0.24 | -0.61 - 0.14 | 0.226 |
| Type of urbanization: rural |  | 0.20 | -0.20 - 0.60 | 0.323 |  | 0.07 | -0.34 - 0.48 | 0.744 |
| Parental education: high school or less (ref: University) |  | -0.01 | -0.35 - 0.32 | 0.931 |  | -0.18 | -0.58 - 0.23 | 0.394 |
| Parental education: college |  | 0.13 | -0.11 - 0.38 | 0.287 |  | -0.07 | -0.35 - 0.21 | 0.635 |
| Age in years (centered) |  | 0.55 | 0.45 - 0.66 | **<0.001** |  | 0.64 | 0.51 - 0.76 | **<0.001** |
| Car ownership: ≥ 2 cars (ref: ≤ 1 car) |  | 0.05 | -0.16 - 0.25 | 0.660 |  | -0.10 | -0.35 - 0.16 | 0.454 |
| Home ownership: yes (ref: no) |  | -0.07 | -0.31 - 0.18 | 0.592 |  | 0.26 | -0.03 - 0.56 | 0.084 |
| **Random Effects** | | | | | | | | |
| σ^2^ |  | 1.772 | | |  | 2.079 | | |
| τ_00, School_ID_ |  | 0.143 | | |  | 0.110 | | |
| N_School_ID_ |  | 37 | | |  | 37 | | |
| ICC_School_ID_ |  | 0.075 | | |  | 0.050 | | |
| Observations |  | 803 | | |  | 658 | | |
| R^2^ / Ω_0_^2^ |  | 0.315 / 0.314 | | |  | 0.260 / 0.258 | | |
| AIC |  | 2800.115 | | |  | 2398.174 | | |
| Deviance |  | 2774.115 | | |  | 2372.174 | | |

B, Unstandardized beta coefficient; CI, confidence interval; p, p-value; SES, Socioeconomic status; σ^2^, variance within cluster groups (schools); τ_00_, variance within the dependent variable between schools; N, Number of schools; ICC, Intraclass correlation coefficient; R^2^ / Ω_0_^2^, pseudo R^2^ estimates; AIC, Akaike information criterion

**Table S3.** Relationship between area-level SES and type of urbanization with weekday outdoor time.

| *Independent variables* | *Dependent variable: weekday outdoor time (≤2 hours (ref) vs. >2 hours)* | | | | | | | |
| --- | --- | --- | --- | --- | --- | --- | --- | --- |
|  |  | Girls | | |  | Boys | | |
|  |  | *Odds Ratio* | *95% CI* | *p* |  | *Odds Ratio* | *95% CI* | *p* |
| **Fixed Effects** | | | | | | | | |
| (Intercept) |  | 0.11 | 0.05 - 0.22 | **<0.001** |  | 0.17 | 0.09 - 0.31 | **<0.001** |
| School SES: low (ref: high) |  | 0.88 | 0.55 - 1.43 | 0.616 |  | 0.95 | 0.63 - 1.42 | 0.795 |
| Site: Vancouver (ref: Ottawa) |  | 1.39 | 0.78 - 2.45 | 0.261 |  | 1.27 | 0.75 - 2.13 | 0.377 |
| Site: Trois- Rivières |  | 1.35 | 0.71 - 2.56 | 0.361 |  | 0.85 | 0.47 - 1.54 | 0.584 |
| Type of urbanization: suburban (ref: urban) |  | 2.08 | 1.19 - 3.62 | **0.010** |  | 0.98 | 0.62 - 1.55 | 0.947 |
| Type of urbanization: rural |  | 2.08 | 1.14 - 3.78 | **0.017** |  | 0.77 | 0.43 - 1.36 | 0.362 |
| Parental education: high school or less (ref: University) |  | 0.87 | 0.44 - 1.75 | 0.703 |  | 1.41 | 0.72 - 2.80 | 0.319 |
| Parental education: college |  | 1.58 | 0.99 - 2.53 | 0.057 |  | 1.04 | 0.63 - 1.70 | 0.887 |
| Age in years (centered) |  | 0.84 | 0.68 - 1.03 | 0.099 |  | 0.93 | 0.76 - 1.15 | 0.494 |
| Car ownership: ≥ 2 cars (ref: ≤ 1 car) |  | 1.07 | 0.70 - 1.64 | 0.768 |  | 0.98 | 0.63 - 1.52 | 0.929 |
| Home ownership: yes (ref: no) |  | 0.80 | 0.49 - 1.29 | 0.356 |  | 1.50 | 0.89 - 2.52 | 0.129 |
| **Random Effects** | | | | | | | | |
| τ_00, School_ID_ |  | 0.126 | | |  | 0.000 | | |
| N_School_ID_ |  | 37 | | |  | 37 | | |
| ICC_School_ID_ |  | 0.037 | | |  | 0.000 | | |
| Observations |  | 800 | | |  | 654 | | |
| Tjur's D |  | 0.045 | | |  | 0.011 | | |
| AIC |  | 726.159 | | |  | 657.741 | | |
| -2 Log-Likelihood |  | 702.159 | | |  | 633.741 | | |
| Deviance |  | 681.485 | | |  | 633.741 | | |

CI, Confidence interval; p. p-value; SES, Socioeconomic status; N, Number of schools; τ_00_, variance within the dependent variable between schools; ICC, Intraclass correlation coefficient; AIC, Akaike information criterion

**Table S4.** Relationship between area-level SES and type of urbanization in weekend day outdoor time.

| *Independent variables* | *Dependent variable: weekend day outdoor time (≤2 hours (ref) vs. >2 hours)* | | | | | | | |
| --- | --- | --- | --- | --- | --- | --- | --- | --- |
|  |  | Girls | | |  | Boys | | |
|  |  | *Odds Ratio* | *95% CI* | *p* |  | *Odds Ratio* | *95% CI* | *p* |
| **Fixed Effects** | | | | | | | | |
| (Intercept) |  | 1.18 | 0.72 - 1.94 | 0.512 |  | 0.82 | 0.44 - 1.54 | 0.537 |
| School SES: low (ref: high) |  | 0.48 | 0.33 - 0.70 | **<0.001** |  | 0.59 | 0.37 - 0.95 | **0.031** |
| Site: Vancouver (ref: Ottawa) |  | 0.67 | 0.44 - 1.04 | 0.073 |  | 0.79 | 0.45 - 1.40 | 0.416 |
| Site: Trois- Rivières |  | 1.66 | 1.00 - 2.75 | **0.048** |  | 2.19 | 1.15 - 4.17 | **0.018** |
| Type of urbanization: suburban (ref: urban) |  | 1.33 | 0.87 - 2.04 | 0.186 |  | 0.77 | 0.44 - 1.33 | 0.346 |
| Type of urbanization: rural |  | 2.27 | 1.41 - 3.64 | **<0.001** |  | 1.36 | 0.74 - 2.49 | 0.318 |
| Parental education: high school or less (ref: University) |  | 1.00 | 0.59 - 1.69 | 0.990 |  | 1.74 | 0.94 - 3.23 | 0.077 |
| Parental education: college |  | 1.46 | 1.00 - 2.15 | 0.052 |  | 1.50 | 0.99 - 2.29 | 0.057 |
| Age in years (centered) |  | 0.79 | 0.67 - 0.93 | **0.004** |  | 1.00 | 0.84 - 1.21 | 0.966 |
| Car ownership: ≥ 2 cars (ref: ≤ 1 car) |  | 0.83 | 0.60 - 1.15 | 0.264 |  | 1.20 | 0.83 - 1.75 | 0.339 |
| Home ownership: yes (ref: no) |  | 0.80 | 0.55 - 1.17 | 0.243 |  | 1.41 | 0.92 - 2.18 | 0.117 |
| **Random Effects** | | | | | | | | |
| τ_00, School_ID_ |  | 0.081 | | |  | 0.212 | | |
| N_School_ID_ |  | 37 | | |  | 37 | | |
| ICC_School_ID_ |  | 0.024 | | |  | 0.061 | | |
| Observations |  | 798 | | |  | 651 | | |
| Tjur's D |  | 0.098 | | |  | 0.119 | | |
| AIC |  | 1061.254 | | |  | 862.499 | | |
| -2 Log-Likelihood |  | 1037.254 | | |  | 838.499 | | |
| Deviance |  | 1015.283 | | |  | 802.565 | | |

CI, confidence interval; p, p-value; SES, Socioeconomic status; τ_00_, variance within the dependent variable between schools; N, Number of schools; ICC, Intraclass correlation coefficient, AIC, Akaike information criterion

**Table S5.** Relationship between area-level SES and type of urbanization with average number of steps per day.

| *Independent variables* | *Dependent variable: steps per day* | | | | | | | |
| --- | --- | --- | --- | --- | --- | --- | --- | --- |
|  |  | Girls | | |  | Boys | | |
|  |  | *B* | *95% CI* | *p* |  | *B* | *95% CI* | *p* |
| **Fixed Effects** | | | | | | | | |
| (Intercept) |  | 11062.80 | 9736.03 - 12389.58 | **<0.001** |  | 13106.26 | 11605.53 - 14606.99 | **<0.001** |
| School SES: low (ref: high) |  | -497.27 | -1544.52 - 549.99 | 0.356 |  | -856.00 | -1997.47 - 285.47 | 0.147 |
| Site: Vancouver (ref: Ottawa) |  | 729.66 | -515.91 - 1975.23 | 0.256 |  | -140.80 | -1528.02 - 1246.43 | 0.843 |
| Site: Trois- Rivières |  | -272.00 | -1602.77 - 1058.76 | 0.690 |  | 549.41 | -937.27 - 2036.10 | 0.472 |
| Type of urbanization: suburban (ref: urban) |  | 205.16 | -1032.49 - 1442.80 | 0.747 |  | 310.01 | -1033.50 - 1653.52 | 0.653 |
| Type of urbanization: rural |  | 43.31 | -1235.78 - 1322.40 | 0.947 |  | 69.00 | -1347.18 - 1485.17 | 0.924 |
| Parental education: high school or less (ref: University) |  | -205.85 | -1114.69 - 702.99 | 0.659 |  | -345.89 | -1671.42 -  979.64 | 0.611 |
| Parental education: college |  | 145.19 | -515.28 - 805.67 | 0.668 |  | -267.08 | -1128.80 - 594.63 | 0.546 |
| Age in years (centered) |  | -13.86 | -291.64 - 263.91 | 0.922 |  | -531.49 | -920.59 - -142.39 | **0.010** |
| Car ownership: ≥ 2 cars (ref: ≤ 1 car) |  | 167.39 | -388.86 - 723.64 | 0.558 |  | 343.16 | -440.42 - 1126.74 | 0.394 |
| Home ownership: yes (ref: no) |  | 167.86 | -494.43 - 830.15 | 0.621 |  | 64.04 | -870.00 -  998.07 | 0.894 |
| **Random Effects** | | | | | | | | |
| σ^2^ |  | 10608389.460 | | |  | 15340797.774 | | |
| τ_00, School_ID_ |  | 1842958.580 | | |  | 1694874.092 | | |
| N_School_ID_ |  | 37 | | |  | 37 | | |
| ICC_School_ID_ |  | 0.148 | | |  | 0.099 | | |
| Observations |  | 684 | | |  | 502 | | |
| R^2^ / Ω_0_^2^ |  | 0.192 / 0.182 | | |  | 0.170 / 0.154 | | |
| AIC |  | 13082.747 | | |  | 9788.602 | | |
| Deviance |  | 13056.747 | | |  | 9762.602 | | |

B, Unstandardized beta coefficient; CI, Confidence interval; p, p-value; SES, Socioeconomic status; σ^2^, variance within cluster groups (schools); τ_00_, variance within the dependent variable between schools; N, Number of schools; ICC, Intraclass correlation coefficient; R^2^ / Ω_0_^2^, pseudo R^2^ estimates; AIC, Akaike information criterion

**Table S6.** Relationship between area-level SES and type of urbanization with average minutes of MVPA per day.

| *Independent variables* | *Dependent variable: MVPA per day* | | | | | | | |
| --- | --- | --- | --- | --- | --- | --- | --- | --- |
|  |  | Girls | | |  | Boys | | |
|  |  | *B* | *95% CI* | *p* |  | *B* | *95% CI* | *p* |
| **Fixed Effects** | | | | | | | | |
| (Intercept) |  | 57.23 | 49.49 - 64.97 | **<0.001** |  | 66.39 | 56.35 - 76.43 | **<0.001** |
| School SES: low (ref: high) |  | -2.63 | -8.74 - 3.48 | 0.402 |  | -3.20 | -10.86 - 4.47 | 0.417 |
| Site: Vancouver (ref: Ottawa) |  | 5.52 | -1.74 - 12.78 | 0.142 |  | 2.26 | -7.06 - 11.59 | 0.636 |
| Site: Trois- Rivières |  | 0.38 | -7.38 - 8.14 | 0.924 |  | 6.71 | -3.23 - 16.65 | 0.191 |
| Type of urbanization: suburban (ref: urban) |  | 2.15 | -5.07 - 9.36 | 0.562 |  | 2.85 | -6.23 - 11.92 | 0.541 |
| Type of urbanization: rural |  | 1.80 | -5.66 - 9.26 | 0.639 |  | 2.33 | -7.15 - 11.82 | 0.631 |
| Parental education: high school or less (ref: University) |  | -0.64 | -5.97 - 4.70 | 0.816 |  | -2.00 | -10.38 - 6.37 | 0.641 |
| Parental education: college |  | 0.43 | -3.45 - 4.31 | 0.829 |  | -1.70 | -7.16 - 3.75 | 0.543 |
| Age in years (centered) |  | -0.50 | -2.13 - 1.13 | 0.547 |  | -4.28 | -6.74 - -1.81 | **0.001** |
| Car ownership: ≥ 2 cars (ref: ≤ 1 car) |  | 0.66 | -2.60 - 3.93 | 0.692 |  | 1.62 | -3.33 - 6.57 | 0.524 |
| Home ownership: yes (ref: no) |  | 0.90 | -2.99 - 4.78 | 0.653 |  | 1.34 | -4.58 - 7.27 | 0.658 |
| **Random Effects** | | | | | | | | |
| σ^2^ |  | 365.479 | | |  | 608.529 | | |
| τ_00, School_ID_ |  | 62.316 | | |  | 83.868 | | |
| N_School_ID_ |  | 37 | | |  | 37 | | |
| ICC_School_ID_ |  | 0.146 | | |  | 0.121 | | |
| Observations |  | 683 | | |  | 500 | | |
| R^2^ / Ω_0_^2^ |  | 0.192 / 0.182 | | |  | 0.191 / 0.175 | | |
| AIC |  | 6044.736 | | |  | 4687.024 | | |
| Deviance |  | 6018.736 | | |  | 4661.024 | | |

MVPA, Moderate-to-vigorous physical activity; B, Unstandardized beta coefficient; CI, Confidence interval; p; P-value; SES, Socioeconomic status; σ^2^, variance within cluster groups (schools); τ_00_, variance within the dependent variable between schools; ICC, Intraclass correlation coefficient; R^2^ / Ω_0_^2^, pseudo R^2^ estimates; AIC, Akaike information criterion
